# Accès aux soins chirurgicaux, obstétricaux, et anesthésiques au Mali: état des lieux pour l’élaboration d’un plan national

**DOI:** 10.11604/pamj.2025.52.112.49160

**Published:** 2025-11-14

**Authors:** Adégné Togo, Ousmane Sy, Arouna Adama Doumbia, Oumar Sangho, Bakary Tientigui Dembélé, Yeya Sadio Sarro, Moussa Samaké, Seydina Alioune Beye, Seydou Pamateck, Souleymane Binke Dembélé, Lassana Kanté, Ghislain Gnimbar Poda, Ibrahima Téguété, Ibrahim Dolo, Youssouf Coulibaly, Alhassane Traoré, Seydou Doumbia

**Affiliations:** 1 Service de Chirurgie Générale, Centre Hospitalier Universitaire Gabriel Touré, Laboratoire de Recherche Appliquée pour le Développement de la Chirurgie et de l'Anatomie, Faculté de Médecine et d'Odonto-Stomatologie - Université des Sciences, des Techniques et des Technologies de Bamako, Bamako, Mali,; 2 Ceped, Université Paris Cité, Université Sorbonne Paris Nord, IRD, Inserm, F-75006 Paris, France,; 3 Organisation Mondiale de la Santé, Bamako, Mali,; 4 Département de Santé Publique, Faculté de Médecine Odonto-Stomatologie, Université des Sciences des Techniques et des Technologies de Bamako, Bamako, Mali,; 5 Hôpital du District de Bamako, Bamako, Mali,; 6 Service Anesthésie Réanimation Clinique Périnatale Mohamed V, Laboratoire de Recherche Appliquée pour le Développement de la Chirurgie et de l'Anatomie, Faculté de Médecine et d'Odonto-Stomatologie - Université des Sciences, des Techniques et des Technologies de Bamako, Bamako, Mali,; 7 Service de Chirurgie, Centre de Référence Commune 5, Laboratoire de Recherche Appliquée pour le Développement de la Chirurgie et de l'Anatomie, Faculté de Médecine et d'Odonto-Stomatologie - Université des Sciences, des Techniques et des Technologies de Bamako, Bamako, Mali,; 8 Service de Chirurgie, Hôpital du District de Bamako, Bamako, Mali,; 9 Agence des États-Unis pour le Développement International, Bamako, Mali,; 10 Direction Générale de la Santé et de l'Hygiène Publique, Bamako, Mali

**Keywords:** Accès, soins chirurgicaux obstétricaux et anesthésiques, plan national du Mali, Assessment, obstetric and anaesthetic surgical care, national plan for Mali

## Abstract

L'élaboration d'un plan national de chirurgie, d'obstétrique et d'anesthésie reste une priorité pour le Mali. Notre objectif était d'évaluer la disponibilité, l'accessibilité, la qualité et le financement des soins chirurgicaux, obstétricaux et anesthésiques, afin de fournir les bases scientifiques pour l'élaboration d'un plan national. C'était une étude transversale, prospective faite de janvier à juin 2023. Toutes les régions du Mali et Bamako ont été incluses. Dans chaque région au moins un district sanitaire a été tiré au sort. Nous avons retenu 25 centres soit 38% des structures publiques. L'enquête a concerné les agents de santé et les patients. Les données ont été analysées sur le logiciel R version 4.5.1. Chaque centre de santé de référence (CSRef) couvrait une aire de santé dont la population variait entre 7913 habitants et 818436 habitants. La durée pour atteindre la structure publique la plus proche offrant les soins chirurgicaux sûrs était de 2 heures pour 50% de la population. La capacité d'accueil totale des structures était de 1343 lits, soit une moyenne de 34 lits pour 100 000 habitants. Tous les 25 centres de santé de référence disposaient d'au moins un bloc opératoire. Le trio de base (chirurgien généraliste, gynécologue-obstétricien, anesthésiste-réanimateur) était disponible dans 14 centres (56%). Le nombre moyen d'actes chirurgicaux annuels était de 135 pour 100 000 habitants. La césarienne, la cure de la hernie, l'appendicectomie et la laparotomie étaient réalisées dans tous les centres. Les fractures ouvertes étaient traitées dans six centres. Les coûts des mêmes actes étaient variables d'un centre à un autre et le taux de couverture d'assurance maladie était de 1 à 25%. Au Mali la chirurgie n'est pas accessible pour la majorité des populations. Les limites sont la distance géographique, la disponibilité de blocs opératoires bien équipés, de spécialistes et le faible taux de couverture d'assurance maladie.

## Abstract (en)

The development of a national plan for surgery, obstetrics, and anaesthesia remains a priority for Mali. Our objective was to assess the availability, accessibility, quality, and financing of surgical, obstetric, and anaesthesia care, to provide the scientific basis for developing a national plan. This was a cross-sectional, prospective study conducted from January to June 2023. All regions of Mali, including the district of Bamako, were included. In each region, at least one health district was randomly selected. We selected 25 centres, representing 38% of public facilities. The survey involved district hospitals, health workers and patients. The data were analysed using R software version 4.5.1. Each hospital of the district covered a health area with a population ranging from 7,913 to 818,436. The time to reach the nearest public facility offering safe surgical care was 2 hours for 50% of the population. The total capacity of the facilities was 1,343 beds, or an average of 34 beds per 100,000 inhabitants. All 25 referral health centres had at least one operating room. The basic trio (general surgeon, obstetrician-gynaecologist, anesthesiologist-resuscitator) was available in 14 centres (56%). The average number of annual surgical procedures was 135 per 100,000 inhabitants. Caesarean section, hernia repair, appendectomy, and laparotomy were performed in all centres. Open bone injuries were treated in six centres. Costs for the same procedures varied from one centre to another, and health insurance coverage rates ranged from 1% to 25%. In Mali, surgery is inaccessible to the majority of the population. Limitations include geographic distance, the availability of well-equipped operating rooms and specialists, and low health insurance coverage rates.

## Introduction

La chirurgie occupe une place importante dans le système de soins. Pourtant en 2014, environ 5 milliards de personnes dans le monde n'avaient pas accès à des soins chirurgicaux, obstétricaux et anesthésiques sûrs, abordables et de qualité dont la majorité en Afrique [1-3]. Selon Shrime *et al*. en 2010 environ 16,9 millions de décès liés au manque de soins chirurgicaux, obstétricaux et anesthésiques ont été enregistrés. Ces décès survenaient majoritairement dans les pays en développement où le nombre de décès liés au manque de soins chirurgicaux dépassait le cumul des décès liés aux 3 pathologies infectieuses qui sont le paludisme, la tuberculose et le VIH [3,4].

Cette surmortalité observée en Afrique serait liée à plusieurs facteurs parmi lesquels, les besoins non couverts en soins chirurgicaux plus importants, la multiplication des conflits armés, la rareté de ressources humaines qualifiées, l'insuffisance de ressources financières [5,6]. Pour relever ces défis, l'Organisation mondiale de la Santé (OMS) recommande l'amélioration de l'accès aux soins chirurgicaux, par l'élaboration et la mise en œuvre d'un plan national de chirurgie, d'obstétrique et d'anesthésique dans tous les pays, afin d'intégrer la chirurgie dans les priorités des systèmes de santé. Ainsi, en 2022, un Symposium international sur le renforcement des systèmes de soins chirurgicaux, obstétricaux et anesthésiques à l'horizon 2030 en Afrique a été organisé à Dakar suggérant la prise en compte de la chirurgie comme une partie essentielle du système de santé et de son équipement [7]. Ces conclusions rejoignaient également celles de l'OMS et de Spiegel *et al*. qui recommandaient aux pays d'élaborer un plan national de chirurgie, obstétrique et anesthésique [8,9].

Au Mali, la recherche et les politiques de santé se sont jusqu'ici concentrées principalement sur les maladies infectieuses et la santé maternelle et infantile [10]. Les auteurs n'ont pas évalué l'accès aux soins chirurgicaux, obstétricaux et anesthésiques. À ce jour, aucune étude n'avait porté sur l'évaluation des infrastructures, des ressources humaines et de l'offre de soins au Mali. Nous avons donc réalisé cette étude afin de combler ce vide en évaluant, pour la première fois à l'échelle nationale, la disponibilité, l'accessibilité, la qualité et le financement des soins chirurgicaux, obstétricaux et anesthésiques, afin de fournir les bases scientifiques nécessaires à l'élaboration d'un plan national adapté au Mali

## Méthodes

**Figure 1:**
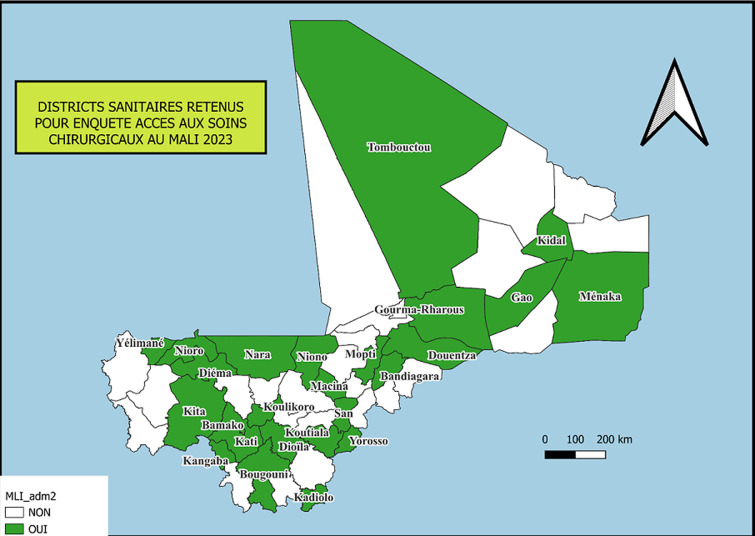
carte du Mali montrant les 25 districts sanitaires retenus pour l'étude

Nous avons réalisé une étude transversale descriptive sur une période de six mois (janvier 2023 à juin 2023). Le cadre de l'étude a couvert l'ensemble du territoire malien, incluant les 19 régions administratives et le district de Bamako. Un échantillonnage stratifié a été réalisé afin d'assurer la représentativité nationale. Dans chaque région, au moins une structure sanitaire publique a été tirée au sort, ce qui a permis d'inclure 25 centres de santé de référence; soit 38% des 65 centres de santé de référence recensés au Mali en 2023 (la Figure 1 montrant les districts sanitaires sélectionnés).

**Critères d'inclusion:** nous avons inclus les centres de santé de référence du secteur public offrant des soins chirurgicaux obstétricaux et anesthésiques.

**Critères de non-inclusion:** les structures inaccessibles pour des raisons sécuritaires ou logistiques (notamment l'absence d'internet empêchant la collecte des données).

**Collecte des données:** la collecte a été réalisée à l'aide de la fiche standardisée de l'OMS pour les hôpitaux de district, validée et utilisée dans plusieurs pays africains pour évaluer les capacités chirurgicales. L'outil a été adapté et numérisé sur la plateforme Kobotoolbox. Un médecin référent (médecin chef ou autre médecin du centre disponible) avec contact téléphonique et email a été identifié pour chaque établissement retenu. Les questionnaires ont été renseignés par les médecins-chefs, chargés SIS (système d'information sanitaire), chirurgiens et autres spécialistes selon les centres de santé de référence sélectionnés. Les questions non comprises ont été traitées par échanges directs entre le coordinateur et les correspondants. Les données sur les coûts, l'offre des soins, la sécurité des soins et les suites postopératoires ont été obtenues auprès des spécialistes et avec l'exploitation des registres des centres. Le niveau de sécurité des actes chirurgicaux et anesthésiques a été évalué par le spécialiste qui pose le geste

**Variables étudiées:** les données collectées portaient sur les cinq axes stratégiques définis par la commission Lancet qui étaient: 1 accès géographique et disponibilité des structures, 2 ressources humaines, 3 infrastructures et équipements, 4 offre de soins chirurgicaux, obstétricaux et anesthésiques, 5 financements, gouvernance et données.

**Analyses des données:** les données ont été importées et analysées sur le logiciel R version 4. Les variables quantitatives ont été exprimées en moyenne, ± écart-type, ainsi qu'en médiane avec extrêmes (maximum et minimum). Les variables qualitatives ont été présentées sous forme de fréquences et de pourcentages. Les figures ont été réalisées avec le logiciel QSIS.

**Considérations éthiques:** la participation des structures à l'étude a été volontaire. Une autorisation a été obtenue auprès du comité scientifique de la Société de Chirurgie du Mali pour réaliser le travail. Les données collectées ne comportaient aucun identifiant personnel et sont demeurées strictement confidentielles. Les auteurs déclarent n'avoir aucun conflit d'intérêt.

## Résultats

Au total, 25 centres de santé de référence répartis dans 19 régions et le district de Bamako ont été inclus, couvrant une population estimée à 9 301 083 habitants.

**Accès géographique et disponibilités des structures:** chaque CSRef couvrait une aire de santé dont la population variait entre 7913 habitants (aire de santé la moins peuplée) et 818436 habitants (aire de santé la plus peuplée). Les patients de chaque aire venaient de divers sites pour les soins dans les structures évaluées. La proportion de population ayant accès en moins de 2 heures de route en voiture était de 1% au grand Nord, 25% au centre et à Kayes et 50% dans la capitale et les régions de Ségou, Sikasso et Koulikoro. La durée pour atteindre la structure publique la plus proche offrant les soins chirurgicaux sûrs était de 2 heures pour 50% de la population. La capacité d'accueil totale des structures était de 1343 lits, soit une moyenne de 34 lits pour 100 000 habitants (les districts sanitaires avec leur nombre de structures et de lits pour 100 000 habitants sont résumés dans le Tableau 1).

**Tableau 1:** districts sanitaires retenus, taille de la population et nombre de lits par 100 000 habitants

Num	Ville	Taille de la population de l’aire de santé	Nombre de CSRef offrant les soins chirurgicaux	Nombre de lits en chirurgie et gynéco/100000 habitants
-	Commune VI	702893	4	6
-	DIEMA	316348	1	6
-	Gao	357720	1	6
-	NIORO	351274	1	7
-	Kidal	7913	1	202
-	Mopti	550056	1	11
-	Meneka	41678	1	43
-	Kadiolo	360310	1	9
-	Kangaba	150644	1	13
-	Macina	351170	1	22
-	Niono	544896	1	10
-	Koutiala	818436	2	5
-	Yorosso	317916	1	6
-	Taoudenit	26721	0	0
-	Gourma Rharous	227970	1	13
-	Ouelessebougou	289731	1	16
-	Tombouctou	184136	1	9
-	Yelimané	263609	1	6
-	Douentza	420982	1	6
-	San	511910	1	8
-	Kita	530956	1	5
-	Nara	373483	1	12
-	Dioila	407702	1	13
-	Bandiagara	480630	1	5
-	Bougouni	711999	2	7
**-**	**TOTAL**	**9301083**	**29**	**446**

**Infrastructures et équipements:** tous les 25 centres disposaient d'au moins un bloc opératoire fonctionnel. Les mêmes blocs étaient utilisés pour la chirurgie des enfants et des adultes, et par toutes les spécialités. Les unités de soins intensifs pour adultes étaient disponibles et fonctionnelles dans 8 centres (32%), les 17 autres n'en disposaient pas. Les unités de soins intensifs pédiatriques étaient fonctionnelles dans 3 centres sur les 25 enquêtés (12%).

**Disponibilité et fonctionnalité des équipements:** l'appareil d'anesthésie était disponible et fonctionnel en continu dans 12 centres (48%), disponible et fonctionnel de manière intermittente (un jour sur deux) dans 6 centres (24%) et absent dans 7 centres (28%). L'oxygène était disponible 24 heures sur 24, tous les jours dans 18 centres (78%), manquait 4 mois sur 12 dans 3 centres (12%). Un aspirateur était disponible et fonctionnel dans tous les centres. Le bistouri électrique était disponible et fonctionnel dans 5 centres sur les 25, soit 20%.

**Figure 2:**
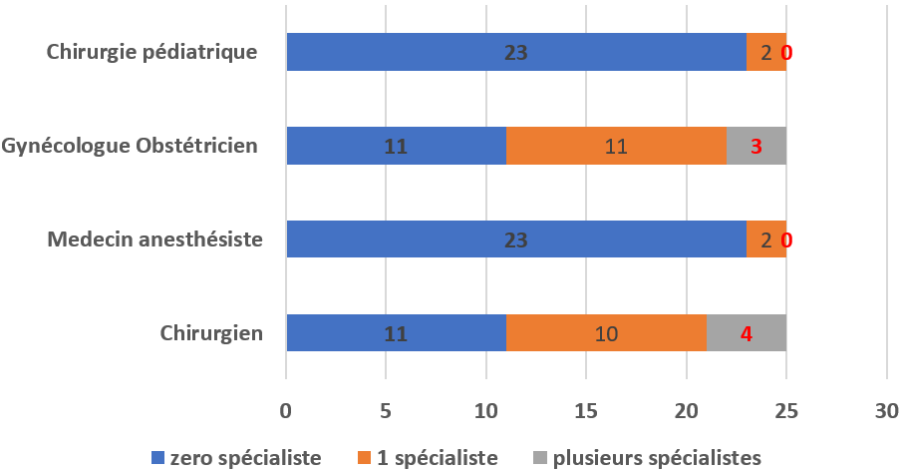
disponibilité et qualité des ressources humaines

**Ressources humaines:** le trio de base (chirurgien généraliste, gynécologue-obstétricien, anesthésiste-réanimateur) était disponible dans 14 centres (56%). Cependant, l'anesthésiste était soit un médecin anesthésiste soit un infirmier anesthésiste. Deux centres disposaient d'un chirurgien pédiatre et d'un traumatologue orthopédiste. Dans 11 centres de santé (44%), les soins chirurgicaux étaient réalisés par des médecins généralistes faisant fonction de chirurgien. Dans sept centres, aucun spécialiste en anesthésie n'était disponible. La qualité des ressources humaines est résumée sur la Figure 2. Les ratios du personnel paramédical se présentaient comme suit: i) infirmier: 1 infirmier pour 7 patients dans 13 centres; 1 infirmier pour plus de 7 patients dans les 12 autres. ii) sage-femme ou infirmière obstétricienne: 1 sage-femme pour moins de 7 femmes enceintes dans 13 entres (56%); 1 sage-femme pour plus de 7 femmes enceintes et/ou parturientes.

### Offres des soins chirurgicaux, obstétricaux et anesthésiques

*Examens complémentaires pour le diagnostic:* la radiographie standard était disponible et fonctionnelle 24 heures sur 24 et tous les jours dans 5 centres (20%), limitée aux heures ouvrables dans 9 centres (36%), absente dans 11 centres (44%). L'échographie était disponible en continu dans 14 centres (56%). Le groupage Rhésus et la numération formule sanguine (NFS) étaient réalisables tous les jours dans 14 centres (16%) ; réalisables que les jours ouvrables dans 4 centres (16%) ; le groupage Rhésus seul était réalisable sans la NFS dans 7 centres (28%). Pour réaliser une transfusion sanguine, le délai moyen d'obtention du sang était < 2 h dans 7 centres (28%); > 2 h dans 18 centres (72%).

**Figure 3:**
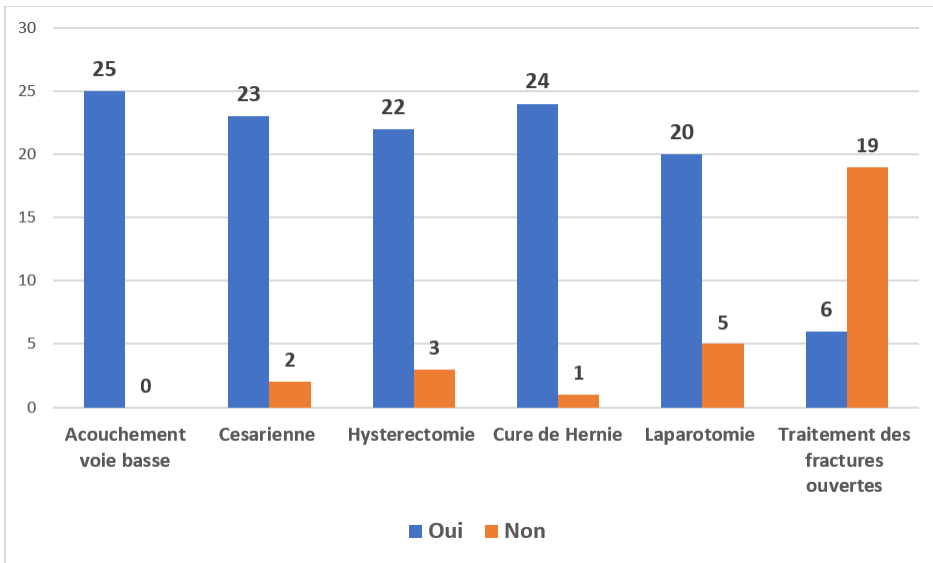
offres de soins chirurgicaux et obstétricaux chez l'adulte dans les 25 structures 2022

**Figure 4:**
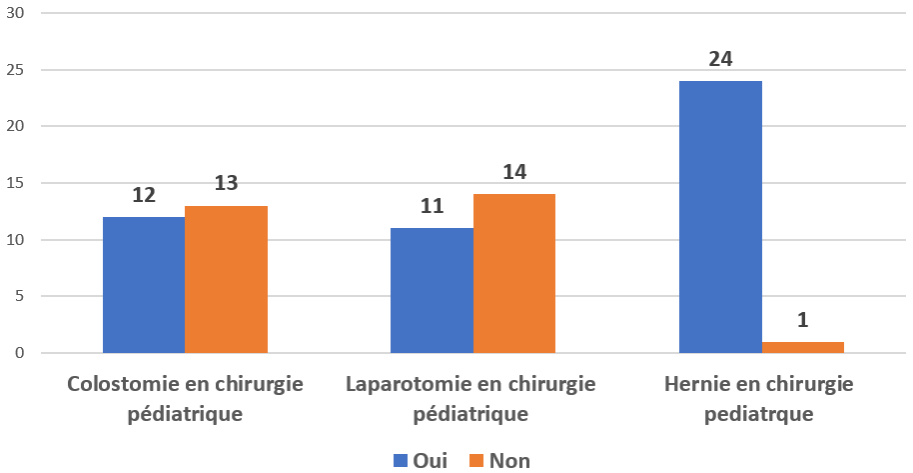
offres de soins chez l'enfant dans les 25 structures en 2022

*Interventions chirurgicales:* i) le nombre moyen d'interventions chirurgicales rapporté à la population de l'aire de santé par centre de santé était de 113 à 150 interventions pour 100 000 habitants par an. ii) type d'actes et capacité de réalisation des centres : les actes chirurgicaux réalisés dans les établissements enquêtés incluaient: 1) la césarienne: réalisable dans 23 centres (92%); 2) l'hystérectomie: réalisable dans 22 centres (88%); 3) la cure de hernie : réalisable dans 24 centres (96%); 4) la laparotomie: réalisable dans 20 centres (80%); 5) fractures ouvertes: réparables dans 6 centres (24%). Sur les 25 centres de santé de référence, 1 centre (4%) ne réalisait que les accouchements par voie basse, 2 centres (8%) ne pouvaient pas faire 2 interventions chirurgicales simultanément, 19 centres (68%) pouvaient réaliser 2 interventions simultanées et trois 3 centres (12%) pouvaient réaliser plus de 2 interventions simultanément. Les Figure 3 et Figure 4 résument les offres de soins chez les adultes et les enfants.

*Actes d'anesthésie réalisables et leur sécurité:* i) anesthésie chez l'adulte: l'anesthésie générale et locorégionale était réalisée chez les adultes dans tous les centres. Elle a été jugée sûre à 76-99% par les praticiens dans 19 centres (76%). ii) anesthésie générale pédiatrique: la réalisation de l'anesthésie générale chez les enfants était possible dans 9 centres et a été jugée sûre à 76-99% par le spécialiste praticien. Elle était jugée sûre dans 4 centres à moins de 50%, elle n'était pas dans les 12 autres centres. La check-list de sécurité de l'OMS était utilisée de façon systématique et correcte (tous les items remplis au moment indiqué) dans 6 centres, de façon incorrecte (tous les items ne sont pas remplis) dans 6 centres, jamais utilisée dans 13 centres.

*Spécificités de la prise en charge des urgences et complications infectieuses:* à l'évaluation de la prise en charge des urgences chirurgicales, obstétricales et des infections postopératoires, sur les 25 centres, 17 avaient une salle d'urgence fonctionnelle tous les jours et 24 heures sur 24 pour les patients provenant de l'extérieur. Nous avons trouvé 19 structures de santé qui disposaient d'un programme de prévention et de contrôle des infections fonctionnelles. Ces programmes permettaient de déterminer le taux d'infections postopératoires et la morbi-mortalité postopératoire.

### Financement, gouvernance et données

**Couverture assurantielle:** les patients disposant d'une assurance maladie représentaient 1-25% des usagers des structures.

*Coûts des soins:* les coûts moyens des différents actes se présentaient comme suit: i) bilan biologique standard: 13 500Fcfa; ii) radiographie: 6 700Fcfa; iii) échographie: 7000Fcfa; iv) actes chirurgicaux: 15 000-25 000Fcfa; v) gouvernance: tous les 25 centres de référence disposaient d'un conseil de gestion dans lequel siège un représentant de la communauté.

**Gestion des données et surveillance épidémiologique:** dans le cadre de la surveillance épidémiologique tous les 25 centres envoyaient les rapports à leur direction régionale de santé. Les données sur le nombre d'actes de chirurgie réalisés et le nombre de décès étaient disponibles dans 18 centres. Pour l'année 2022, sur les 8188 malades opérés, la mortalité postopératoire au cours des 30 jours suivant la chirurgie a été de 0,7% soit 61 décès.

## Discussion

**Forces et limites:** cette enquête nationale constitue la première évaluation exhaustive des capacités chirurgicales, obstétricales et anesthésiques au Mali, couvrant toutes les régions. Elle se distingue par son caractère prospectif et par l'utilisation d'outils validés de l'OMS. Toutefois, elle présente des limites, notamment l'exclusion des structures privées et des hôpitaux nationaux de référence, ce qui pourrait conduire à une sous-estimation du volume chirurgical. Un plan national de chirurgie, d'obstétrique et d'anesthésie peut être élaboré sur la base de ces premiers résultats qui seront complétés par les données des structures privées et celles des hôpitaux nationaux.

**Accès géographique et disponibilité des structures:** l'accessibilité aux structures offrant les soins chirurgicaux est une priorité selon les recommandations du symposium de Dakar. Pour une population de 9 301 083 habitants nous observons une grande inégalité d'accès géographique aux structures de santé offrant les soins chirurgicaux dans les régions et cercles du Mali. La moitié de la population faisait plus de 2 heures de route pour atteindre la structure de santé publique la plus proche pour avoir les soins chirurgicaux obstétricaux et anesthésiques. Comme nous, l'inégalité et les difficultés liées à l'accessibilité géographique des structures de santé en Afrique ont été décrites par Sidiki *et al*. au Mali, Blanford *et al*. au Niger et Levesque *et al*. [11-13]. Pour atteindre l'objectif fixé à Dakar (80% de la population à moins de 2 heures d'une structure chirurgicale d'ici 2030), il sera nécessaire de renforcer le maillage sanitaire et d'améliorer le système de transport et de référence comme le recommandaient ces auteurs.

**Ressources humaines:** le problème des ressources humaines se pose en termes de quantité et de qualité. Les évaluations faites dans l'annuaire statistique concernent tous les agents de santé. Nous avons constaté une insuffisance voire une rareté de spécialistes en dehors des capitales. Dans 11 centres sur 25 il n'y avait ni chirurgien ni gynécologue-obstétricien. Cette insuffisance est encore plus grave en ce qui concerne les spécialités comme la chirurgie pédiatrique, l'orthopédie-traumatologie et l'anesthésie-réanimation. En l'absence de chirurgien, les soins chirurgicaux dans ces zones étaient réalisés par les médecins généralistes. Cette pratique est rapportée dans plusieurs travaux africains [14-16]; Sani *et al*. au Niger et Linden *et al*. en Ouganda proposaient une courte formation spéciale de trois à six mois des médecins généralistes pour améliorer la qualité des services offerts par ces médecins qui sont en zones rurales. Cette formation serait une alternative qui permettra aux hôpitaux des zones rurales de disposer, dans un délai court, d'un personnel permanent, qualifié pour la réalisation des actes de chirurgie pour couvrir les besoins chirurgicaux gynéco-obstétricaux et anesthésiques [14,15].

**Offre de soins chirurgicaux, obstétricaux et anesthésiques:** selon l'OMS, pour 100 000 habitants il faut un objectif de 5000 interventions chirurgicales [3]. Nous rapportons une moyenne annuelle de 113 à 150 interventions pour 100 000 habitants, ce qui est très loin des projections de l'OMS. Certes nos chiffres pourraient être sous-estimés car les interventions chirurgicales réalisées dans les centres privés n'ont pas été comptabilisées. Il est alors urgent de mettre en place une base de données enregistrant toutes les interventions chirurgicales pour mieux évaluer les besoins non couverts en chirurgie dans nos pays. Selon les estimations chaque année, 143 millions d'interventions chirurgicales supplémentaires sont nécessaires dans les pays à revenu faible ou intermédiaire pour sauver des vies et prévenir les handicaps. Dans les pays à faible revenu, neuf personnes sur 10 n'ont pas accès à une anesthésie et à des soins chirurgicaux sûrs et d'un coût abordable [2,3,9].

**Financement, gouvernance et données:** le coût est un élément important pour l'accès aux soins chirurgicaux. Dans le cadre du diagnostic, les examens biologiques et l'imagerie sont très utiles et leurs coûts sont à considérer. Nous rapportons une grande variation du coût des mêmes actes et des examens, d'une structure publique à une autre. Diallo M à Dakar rapportait une variation des coûts selon l'évolution de la maladie et aussi selon les services et les types de traitement [17].

Le taux de couverture d'assurance des malades était très faible entre 1 et 25% selon les centres. Cette faible couverture était rapportée aussi par Boidin *et al*. [18]. Selon Alkire *et al*. l'accès aux soins étant conditionné à la capacité de financer les frais de chirurgie, le système de santé publique doit évoluer vers la participation communautaire et le recouvrement des coûts pour les soins chirurgicaux [5,13].

## Conclusion

Ce premier état des lieux montre qu'au Mali l'accès aux soins chirurgicaux, obstétricaux et anesthésiques est limité par l'accessibilité géographique difficile des structures, l'insuffisance d'équipements adéquats dans les blocs, l'insuffisance de spécialistes et le faible taux de couverture d'assurance maladie pour les malades. Un travail incluant les structures publiques et privées offrant les soins chirurgicaux, obstétricaux et anesthésiques permettrait de faire une évaluation précise des besoins non couverts. Un plan national de chirurgie, d'obstétrique et d'anesthésie peut être élaboré sur la base de ces premiers résultats qui seront complétés par les données des structures privées et celles des hôpitaux nationaux.

### Etat des connaissances sur le sujet

- Au Mali, l'existence des normes et procédures et les référentielles dans les différentes spécialités est connue;
- L'état des lieux de l'accès aux soins pour les pathologies infectieuses;
- Le système de référence est connu.

### Contribution de notre étude à la connaissance

- L'accessibilité des structures offrant les soins chirurgicaux obstétricaux et anesthésiques au Mali et le rapport nombre de lits en chirurgie par 100 000 habitants;
- Les offres de soins et leur accessibilité dans les hôpitaux de district et le nombre de procédure chirurgicale par 100 000 habitants par an;
- La disponibilité des ressources humaines et leur qualité dans les hôpitaux de district et l'accessibilité financière des soins.

## Remerciements

Nous remercions Diallo Mamadou, chirurgien du centre de santé de référence, commune 6, et Guiré Abdou, chirurgien du centre de santé de référence, commune 4.
